# The potential immunological mechanisms of gut microbiota dysbiosis caused by antibiotics exacerbate the lethality of influenza viruses

**DOI:** 10.1080/19490976.2025.2609451

**Published:** 2026-01-02

**Authors:** Jianing Zhu, Zihang Huang, Ying Lin, Jie Zhu, Rui Min, Zibo Wan, Yuting Chen, Jianwen Zhu, Li Xing, Sheng Li, Chinasa Valerie Olovo, Xiaoquan Wang, Guocai Li, Pinghu Zhang

**Affiliations:** a Key Laboratory of the Jiangsu Higher Education Institutions for Integrated Traditional Chinese and Western Medicine in Senile Diseases Control, School of Traditional Chinese Medicine, Faculty of Medicine, Yangzhou University, Yangzhou, China; b Jiangsu Key Laboratory of Zoonosis, Jiangsu Co-Innovation Center for Prevention and Control of Important Animal Infectious Diseases and Zoonoses, Yangzhou University, Yangzhou, Jiangsu, China; c School of Basic Medical Sciences & School of Public Health, Faculty of Medicine, Yangzhou University, Yangzhou, China

**Keywords:** Influenza, lung–gut Immune axis, antibiotics, gut microbiota

## Abstract

**Background:**

Antibiotics are not recommended to treat influenza A virus (IAV). However, antibiotic misuse for IAV persists worldwide. How to scientifically use antibiotics for IAV-infected patients remains a considerable challenge.

**Results:**

Here, we investigated the impact of antibiotics on viral pathogenicity, pulmonary-intestinal antiviral immunity, and antiviral drug efficacy. Our findings indicated that antibiotic intervention exacerbated IAV-caused mortality and lung injury in mice, manifested as increased mortality rates, shortened survival time, aggravated pulmonary injury, and excessive inflammatory responses. Furthermore, antibiotic pretreatment significantly diminished the efficacy of antivirals. Metagenomic sequencing revealed that antibiotics reduced the diversity and abundance of beneficial gut microbiota, including *Lactobacillus* and *Bifidobacterium*, while promoting the proliferation of pathogenic bacteria such as *Klebsiella pneumoniae* and *Escherichia coli*. Mechanistically, antibiotic intervention exacerbated IAV-caused excessive inflammatory responses by the blockage of pulmonary-intestinal antiviral immune pathways, which were caused by the upregulation of PKR, RIG-I, ISG15, and TRIM25 levels while downregulating IPS-1 mRNA levels. However, it is noteworthy that the combination of antibiotics and antiviral drugs effectively offset the adverse effects of antibiotic pretreatment on influenza mortality by upregulating IPS-1 levels and partially restoring pulmonary-intestinal immune homeostasis.

**Conclusions:**

Pulmonary-intestinal immune homeostasis imbalance caused by antibiotic misuse can not only markedly exacerbate the lethality of IAV, but also significantly attenuate the efficacy of antiviral drugs. A mechanistic study confirmed that gut microbes dysbiosis caused by antibiotic pretreatment exacerbates the homeostasis imbalance of host antiviral immunity by blocking the RIG/MDA5/IPS-1 antiviral signaling pathway. However, combination therapy with antibiotics and antivirals effectively reversed the fatal outcome exacerbated by antibiotic pretreatment. Collectively, our findings not only provide a scientific explanation from the perspective of antiviral immunity as to why antibiotics should not be arbitrarily used to treat viral infections but also lay the scientific foundation for the rational clinical use of antivirals and antibiotics for treating influenza.

## 1. Introduction

Influenza is an acute respiratory disease caused primarily by influenza A virus (IAV). Annually, approximately 5 million people worldwide are infected with IAV, with at least 500,000 fatalities.^1^^,^^2^ IAV, a member of the Orthomyxoviridae family, is an RNA virus classified into different subtypes based on variations in hemagglutinin (HA) and neuraminidase (NA) surface proteins. To date, 18 HA subtypes and 11 NA subtypes have been identified.^3^ Owing to the high mutation rate of IAV, new epidemic strains frequently emerge and spread. Furthermore, genetic reassortment and mutation enable the virus to cross species barriers,^4^ contributing to its persistence as a significant global public health threat. Seasonal influenza predominantly affects vulnerable populations, including infants, the elderly, and individuals with compromised immune systems. Moreover, the severity of IAV infection is closely associated with the status of the host's innate immune response.^5^

Antibiotics have been effectively developed and widely used to treat bacterial infections.^6^ However, because antibiotics specifically target bacteria, they are generally not recommended for treating viral infections. The use of antibiotics is typically reserved for managing secondary bacterial infections that may complicate viral illnesses, such as influenza, potentially worsening symptoms or prolonging disease duration.^7^ Despite these guidelines, the antibiotic misuse for viral respiratory infections remains a widespread global issue, contributing to antimicrobial resistance.^8^^,^^9^ Recent surveillance reports have indicated that a substantial fraction of antibiotic prescriptions are unnecessary. For example, Fleming-Dutra et al. found that approximately 30% of outpatient antibiotic prescriptions in the United States were unnecessary,^10^ and Hersh et al. estimated that about 28%–50% of outpatient antibiotic use may be inappropriate or unnecessary in various settings.^11^ Moreover, owing to bacterial evolution and the overuse of antibiotics, the resistance of *Staphylococcus aureus* has gradually increased, leading to a rise in MRSA infection rates worldwide and making clinical anti-infective treatment of MRSA more difficult.^12^^,^^13^ It is estimated that antibiotic-resistant infections are currently responsible for approximately 700,000 deaths worldwide each year.^14^ In addition, antibiotic resistance or antimicrobial resistance in livestock is also a growing global concern that severely threatens both human and animal health. The overuse and misuse of antibiotics in livestock production have resulted in an increased propensity for the development of AMR bacterial strains in animals, which can be spread to humans through the consumption of contaminated animal products, direct contact, or environmental exposure.^15^

However, some clinical studies have reported that the coadministration of antiviral drugs and antibiotics seems to have better efficacy than antiviral drugs alone, suggesting that patients with influenza may benefit from such combination therapy. Etsuhisa Takahashi et al. reported that the macrolide antibiotic clarithromycin enhances the induction of anti-influenza A virus secretory IgA (sIgA) in the airways of H1N1-infected mice and restores the diminished antiviral sIgA levels observed in oseltamivir (OSV)-treated mice to those of controls.^16^ Lee Chien-Wei et al. conducted a single-blind study in patients aged 1–18 y hospitalized with influenza and found that, compared with antiviral monotherapy, combination therapy with antibiotics and antivirals resulted in a faster decline in viral titers, with significant reductions observed as early as 1–3 d after treatment initiation.^17^ Nonetheless, the rationale for antibiotic use in viral infections remains poorly understood and controversial. Furthermore, the potential synergistic or additive effects of combining anti-influenza drugs with antibiotics require further investigation, as the underlying mechanisms and clinical implications have not yet been fully elucidated.

Recent studies have highlighted the critical role of the lung–gut immune axis in modulating host resistance to respiratory viral or bacterial infections. The gut microbiota may affect the antiviral immunity of the lungs through various approaches.^18^ First, because the lung and gut originate from the common mucosal immune system (CMIS), disruption of pulmonary homeostasis may lead to the translocation of the gut microbiota to the lungs.^19^ For example, Dusana Popovic reported that in a mouse model of pulmonary infection caused by fungal pathogens, 41.8% of the new bacterial species that migrated to the lungs were also present in the feces. Moreover, this migration occurred only after the onset of infection.^20^ Second, metabolic products derived from the gut microbiota by entering the bloodstream may affect the antiviral immune response of the lungs. For example, short-chain fatty acids (SCFAs) and indole compounds derived from the gut may exert anti-inflammatory effects in the lungs through various mechanisms.^21-23^ Short-chain fatty acids (SCFAs) as key metabolites produced by the gut microbiota, have a significant impact on both host gut health and systemic metabolism.^24^ In the lungs, SCFAs are utilized by pulmonary immune cells to regulate local immune responses, whereas in the gut, SCFAs can regulate the function of intestinal macrophages to inhibit the proliferation of pathogenic microorganisms by promoting the production of protective regulatory T cells (Tregs), inflammatory cytokines, and the development of dendritic cell.^25^ Experimental studies have confirmed that the administration of vancomycin in mice alters the composition and abundance of the gut microbiota, exacerbating symptoms of allergic airway disease and reducing SCFA production. Conversely, supplementing SCFAs can effectively mitigate this adverse reaction caused by vancomycin and reduce disease severity.^26^ Third, gut-activated immune cells can function systemically by entering the thoracic duct through the lymphatic system and circulating throughout the body.^27^ For example, intrinsic lymphoid cells (ILCs) are a population of antigen receptor-negative immune cells residing on mucosal surfaces that play a critical role in maintaining tissue homeostasis and initiating immune responses. Despite their low abundance in circulation, they exhibit high enrichment within mucosal barriers such as the gut and airways. In pulmonary immune responses, activated intestinal type 2 innate lymphoid cells (ILC2) can utilize lymphocyte function-associated antigen to migrate from the intestine to the lungs, thereby triggering airway inflammation. Huang et al. revealed that ILC2 cells can respond to inflammatory signals and subsequently migrate from the intestine to other organs, including the lungs.^26^ Hu et al. reported that gut microbiota-derived GlcNAc modulates NK cells to protect the host from IAV infection,^28^ further supporting the widespread regulatory role of the gut microbiota in immune function. Fourth, the gut microbiota can influence the host's cytokine profile via multiple pathways, thereby modulating immune responses in distant tissues.^29^ For example, TLR4 recognizes lipopolysaccharides from Gram-negative bacteria, triggering inflammation and leading to the production of cytokines such as TNF-α, IL-1β, and IL-6.^30^ Maschirow et al. also reviewed microbiota-dependent regulation of lung immunity, highlighting the gut–lung axis as a key pathway in which microbial signals modulate cytokines such as IL-22 and activate innate lymphoid cells, which are essential for epithelial repair and barrier maintenance.^31^ Together, these mechanisms demonstrate that the gut microbiota exerts a multifaceted influence on lung antiviral immunity, reinforcing the importance of the lung–gut axis in shaping the host's response to respiratory viral infections.

Given the intrinsic crosstalk between the lung and gut immune systems, antibiotic-induced gut microbiota dysbiosis may impair host immunity against respiratory viral infections. For example, Peng et al. treated mice with antibiotics for two weeks prior to inoculation with influenza A virus and found that antibiotic-induced gut dysbiosis affected the expression of miRNAs in the lungs, thereby interfering with the host's antiviral immunity.^32^ In addition, adverse effects of antibiotics can lead to dysbiosis of the intestinal flora, which can affect the outcome of pulmonary infections. Marvin et al. revealed that the gut dysbiosis caused by vancomycin-clonidine severely impaired monocyte/dendritic cell progenitor cells and lung immunity, exacerbating *Pseudomonas aeruginosa* lung infections in *P. aeruginosa*-infected mice model, suggesting that gut microbiota dysbiosis significantly affects antiviral immunity.^33^ Moreover, early use of antibiotics during viral infections can disrupt the gut microbiota, weakening the host's immune defenses against influenza and exacerbating lung damage. Konjar et al. reported that a healthy gut microbiota may increase the body's ability to generate CD8^+^ T cells.^34^ In contrast, antibiotic intervention increases susceptibility to any virus controlled by T-cell immunity, leading to severe infections or even death.^35^^,^^36^ Additionally, other studies have confirmed that gut microbiota-driven regulatory T cells (Tregs) migrate to sites of organ injury to exert immunotherapeutic effects, whereas antibiotic pretreatment may disrupt the gut microbiota and impair the healing response of injured organs.^37^ Although these studies partially explain the impact of antibiotic-induced gut dysbiosis on host antiviral immunity through the regulation of innate immune cells, the immunological mechanisms driving the disruption of lung–gut antiviral immune homeostasis remain poorly understood. Moreover, it is still unclear whether antibiotic-associated alterations in the gut microbiota influence the efficacy of antiviral agents. To better understand why antibiotics are unsuitable for treating influenza, we first employed an H1N1-infected mouse model to investigate how antibiotic-induced gut dysbiosis influences viral lung injury and the efficacy of an antiviral drug (oseltamivir). We then further examined the underlying immune mechanisms by which disruption of lung–gut immune homeostasis impairs host antiviral defenses against the influenza A virus. These findings aim to provide a scientific basis for the rational use of antibiotics during influenza infection.

## 2. Materials and methods

### 2.1. Reagents

Ampicillin (A430258-0200), vancomycin (A414413-0050), neomycin sulfate (A430130-0005), and metronidazole (A429689-0005) were purchased from Sangon Biotech (Shanghai) Co., Ltd. Oseltamivir phosphate (Batch No. 0372201024) was produced by Yichang Changjiang Pharmaceutical Co., Ltd. The antibiotic suspension (7.5 g/L ampicillin, 3.725 g/L vancomycin, 7.5 g/L neomycin sulfate, 7.5 g/L metronidazole, and 2‰ sweetener) was prepared as previously described.^38^ The RNA isolator (R401-01-AA), HiScript II 1st Strand cDNA Synthesis Kit (R211-01), and ChamQ Universal SYBR qPCR Master Mix (Q711-02) were purchased from Vazyme Biotech Co., Ltd. (Nanjing, China). A stool genomic DNA extraction kit (EasyPure® Stool Genomic DNA Kit (EE301-01)) was obtained from TransGen Biotech Co., Ltd. (Beijing, China).

### 2.2. Mice

Specific pathogen-free (SPF)-grade female (Institute of Cancer Research, ICR) mice weighing 14–15 g (4–5 weeks old) were purchased from the Comparative Medicine Center of Yangzhou University. The mice were housed in an IVC system under a 12-h light/dark cycle at a temperature of 22 °C ± 2 °C and a wet cycle of 50%–70%. The animal experiment protocol was approved by the Animal Experiment Ethics Committee of Yangzhou University (Approval No: 2024070682). All surgical procedures were performed under anesthesia, and every effort was made to minimize pain, distress, and mortality.

### 2.3. Strain source

The mouse-adapted H1N1 influenza virus strain (A/FM/1/47, FM1) was provided by the Key Laboratory of Animal and Poultry Infectious Diseases, Ministry of Agriculture, Yangzhou University. All the animal experiments were conducted in biosafety laboratories at Yangzhou University. The median lethal dose (LD_50_) of the virus was determined according to the following method. Ten-fold dilutions of viral allantoic fluid were prepared using PBS, generating six dilution gradients: 10^−1^, 10^−2^, 10^−3^, 10^−4^, 10^−5^, and 10^−6^. Thirty mice were randomly divided into 6 groups. Each group of mice was infected intranasally under light ether anesthesia with a total of 40 μl of the corresponding dilution of FM1 into both nostrils. Following inoculation, the body weights and deaths of all the groups were recorded for 15 consecutive days. The LD_50_ of the FM1 virus in ICR mice was calculated via the Reed‒Muench method.

### 2.4. Animal experimental design

#### 2.4.1. The influence of different antibiotic intervention modes on the mortality of IAV-infected mice

**Figure 1.**
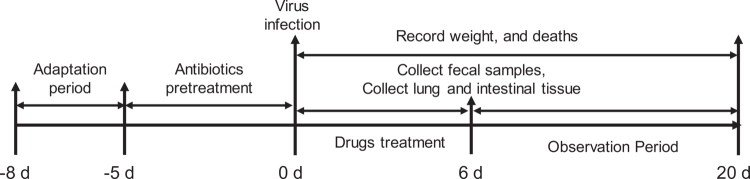
Experimental flowchart of animal experimental design.

The experiment was performed according to the flowchart in Figure 1. After adaptive feeding for 3 d, 72 ICR mice were randomly divided into nine groups (8 mice/group), including the control group, mock group, antibiotic pretreatment group (Antibiotics Pre), antibiotic treatment group (Antibiotics Tre), antibiotic pretreatment and treatment group (Antibiotics Pre + Tre), oseltamivir treatment group (Ose Tre), antibiotic pretreatment plus oseltamivir treatment group (Antibiotics Pre + Ose Tre), antibiotic treatment plus oseltamivir treatment group (Antibiotics + Ose Tre), and antibiotic pretreatment plus antibiotics and oseltamivir treatment group (Antibiotics Pre and Tre + Ose Tre). The Antibiotics Pre groups received antibiotics by gavage at a dose of 0.2 mL/mouse daily for 5 d before virus infection, while the other groups received an equal volume of sterile water by gavage. On day 0, all groups except the control group were intranasally infected with 5LD_50_ H1N1 influenza virus suspension in 40 µL of PBS. Two hours post-infection, the groups designated for antibiotic treatment continued receiving antibiotics for six consecutive days. Eight hours after infection, the groups assigned to the antiviral treatment received oseltamivir (10 mg/kg) once daily for 6 d. The other groups received an equal volume of sterile water for 6 d. Body weight and mortality were recorded daily for 15 d. On day 5 post-infection, fecal samples from each group were collected and stored at −80 °C for subsequent gut microbiota analysis. To further investigate the impact of antibiotic pretreatment on IAV-induced mortality, an additional experiment was conducted using three commonly used clinical antibiotics: metronidazole, azithromycin, and norfloxacin. After adaptive feeding for 3 d, 80 ICR mice were randomly assigned to 10 groups (8 mice/group), including the virus-infected group and two concentrations (a high dose is equivalent to twice the clinical dose, and a low dose is equivalent to once the clinical dose) for each antibiotic. Each antibiotic was administered by gavage daily for 5 consecutive days prior to viral infection, and then were intranasally inoculated with 5LD_50_ H1N1 influenza virus as described above. Body weight was monitored daily for 15 d post-infection, and the relative weight change (%) was calculated based on the weight at day 0. The survival rate was recorded daily. The lung weight/body weight ratio (100%) was measured. All the data were recorded and are expressed as the mean ± SEM. The mice were euthanized if their body weight decreased >25% relative to baseline or upon reaching any other predefined humane endpoint. The data are expressed as the mean ± SEM.

#### 2.4.2. Influence of different antibiotic intervention strategies on IAV-induced lung injury

To determine how different antibiotic intervention strategies influence lung injury, we conducted a second animal experiment. Following the flowchart in Figure 1, 54 female ICR mice were randomly divided into nine groups (6 mice per group), as described in Section 2.4.1. The administration procedures were the same as those described in Section 2.4.1. On day 6 post-infection, mouse blood was collected from the retro-orbital sinus to obtain serum after anesthesia with isoflurane. Fecal samples from each mouse were collected for gut microbiota analysis. Lung and intestinal tissues were collected, and the lung/body weight ratio of each mouse was recorded. The right lung lobe was fixed in 10% formalin solution for histopathological analysis, while the remaining lung tissue was homogenized for cytokine and viral copy analysis. Segments of the duodenum and distal colon were collected and homogenized, and total RNA was extracted to analyze relevant signaling pathways and inflammatory factors.

#### 2.4.3. Preparation and administration of fecal microbiota transplant (FMT)

Fecal pellets collected from healthy control mice were resuspended in sterile phosphate-buffered saline (PBS) at a ratio of 1 g of feces per 10 mL of PBS. The suspension was homogenized and centrifuged at 900 × *g* for 3 min at 4 °C. The resulting supernatant was collected, aliquoted, and stored at −80 °C within 30 min to minimize environmental effects on the microbial community.^39^^,^^40^

A total of 24 mice were randomly allocated into three groups (*n* = 8 per group) and infected as described in Section 2.4.1. Mice in the FMT group received 200 µL of thawed fecal supernatant by oral gavage once daily for 7 d, while the other two groups received an equivalent volume of sterile PBS for 7 d.

### 2.5. Gut microbiota analysis

#### 2.5.1. Microbial genomic DNA extract

Total microbial genomic DNA was extracted from feces samples using the FastPure Stool DNA Isolation Kit (MJYH, Shanghai, China) following the manufacturer's protocol. DNA quality and concentration were assessed via 1.0% agarose gel electrophoresis and a NanoDrop® ND-2000 spectrophotometer (Thermo Scientific Inc., U.S.A.), with aliquots stored at −80 °C until analysis. The V3–V4 hypervariable region of the bacterial 16S rRNA gene was amplified using primers (5-ACTCCTACGGGAGGCAGCAG-3) and R (5-GGACTACHVGGGTWTCTAAT-3) in a T100 Thermal Cycler (Bio-Rad, U.S.A.). Each 20 μL PCR reaction contained 4 μL of 5 × FastPfu buffer, 2 μL of 2.5 mM dNTPs, 0.8 μL of each primer (5 μM), 0.4 μL of FastPfu polymerase, 10 ng of template DNA, and ddH_2_O to volume. The amplification conditions included initial denaturation at 95 °C (3 min), 27 cycles of 95 °C (30 s), 55 °C (30 s), 72 °C (45 s), a final extension at 72 °C (10 min), and a hold at 4 °C. Triplicate PCR products were purified from 2% agarose gels and quantified using a Synergy HTX system (Biotek, U.S.A.).

#### 2.5.2. Illumina sequencing

Purified amplicons were pooled in equimolar amounts and subjected to paired-end sequencing (2 × 300 bp) on an Illumina NextSeq 2000 platform (Illumina, San Diego, CA, U.S.A.) following the manufacturer's protocols. All the experimental procedures were performed by Majorbio Bio-Pharm Technology Co., Ltd. (Shanghai, China). The raw sequencing reads were deposited into the NCBI Sequence Read Archive (SRA) database (Accession Number: PRJNA1274617).

#### 2.5.3. Data processing

Raw FASTQ files were demultiplexed using custom Perl scripts, quality-filtered with FASTP (v0.19.6), and merged via FLASH (v1.2.11) under these criteria: (1) PE300 reads were truncated at sites with average quality scores < 20 (50-bp sliding window), with truncated reads < 50 bp or containing ambiguous bases discarded; (2) Sequences with >10 bp overlaps were assembled (≤20% mismatch tolerance); (3) Samples were demultiplexed via exact barcode matching with ≤2 nucleotide primer mismatches. The quality-controlled sequences were clustered into operational taxonomic units (OTUs) at 97% similarity using USEARCH v11, and the most abundant sequence per OTU was selected as a representative. Chloroplast sequences were systematically removed before rarefaction (20,000 reads/sample; retained Good's coverage > 99%). Taxonomic annotation was performed with RDP Classifier (v2.13) against the SILVA v138 16S rRNA database (confidence threshold: 0.7). Metagenomic functions were predicted via PICRUSt2 through three workflows: HMMER-based sequence alignment, EPA-ng/GAPPA-mediated phylogenetic placement, and CASToR-driven 16S gene copy normalization.^41^ Metabolic pathway reconstruction was performed through a two-stage analytical workflow: (1) Gene family abundance profiles were predicted using MinPath v1.4 with default stringency parameters; (2) functional annotations were systematically mapped to Kyoto Encyclopedia of Genes and Genomes (KEGG) orthology identifiers. All bioinformatic procedures strictly followed the PICRUSt2 computational framework, including its embedded quality control protocols for estimating pathway reliability. The correlations between gut microbes at the genus level and immune factors were evaluated by Spearman's rank correlation coefficient. Statistically significant correlations (*p* < 0.05) are highlighted with asterisks in the heatmap plot.

### 2.6. RT-qPCR

Lung and intestinal tissues were finely cut and accurately weighed to 50 mg. Magnetic beads and 1 mL of RNA isolation reagent were added, and the mixture was homogenized at 4 °C for 3 minutes, with three cycles of grinding to ensure complete homogenization of the lung tissues. Then, 200 μl of chloroform was added, thoroughly mixed, and centrifuged at 12,000 rpm for 10 min at 4 °C. The upper aqueous phase was carefully collected, and an equal volume of isopropanol was added. After mixing, the solution was centrifuged again at 12,000 rpm for 10 min at 4 °C. The supernatant was discarded, and the RNA pellet was washed once with 75% ethanol, followed by centrifugation at 12,000 rpm for 10 min at 4 °C. After the ethanol was removed and allowed to evaporate completely, 100 μl of DEPC-treated water was added to dissolve the RNA. A Nanodrop spectrophotometer was used to quantify the extracted RNA, and 3 μg of total RNA was used for reverse transcription with the following specific protocol: prepare a reaction mixture in an RNase-free centrifuge tube by combining 2  μl of RNase-free ddH_2_O, 1 μl of oligo (dT)23 VN (50 μM), and 3 μg of total RNA. The mixture was heated at 65 °C for 5 min, then rapidly cooled on ice and allowed to sit for 2 min. Next, 10 μl of 2 × RT Mix and 2 μl of HiScript II Enzyme Mix were added to the reaction mixture. Gently mix using a pipette. The mixture was incubated at 25 °C for 5 min, followed by incubation at 50 °C for 45 min, and then the reverse transcriptase was inactivated by heating at 85 °C for 2 min before storing at −20 °C for future use. In the qPCR tubes, 10 μl of 2 × ChamQ Universal SYBR qPCR Master Mix, 0.4 μl of Primer 1 (10 μM), 0.4 μl of Primer 2 (10 μM), and 0.4 μl of template DNA were added, then bring the total volume to 20 μl with ddH_2_O. The PCR was conducted using ChamQ Universal SYBR qPCR Master Mix (Vazyme, China) under the following conditions: 5 min at 95 °C, followed by 35 cycles of 10 s at 95 °C and 30 s at 60 °C, concluding with a melting curve analysis. The primers were synthesized by GenScript (Nanjing, China), and the primer sequences are presented in Supplementary Table 1. The efficiency and gene stability were first confirmed via PCR and DNA agarose gel electrophoresis, and then the homogeneity of the amplification products was analyzed via melting curve analysis.

### 2.7. Cytokines examination

Measurement of mouse serum cytokines (IFN-γ, IL-10, IL-6, TNF-α, IP-10, and MCP-1) was performed using magEasyQPlex Mouse 6-plex Flow Assay Kit (CLEM006, Laizee Biotech) on an Attune NxT Flow Cytometer. Briefly, 25 μL serum samples were incubated for 2 h with magnetic mixture bead sets, which were uniquely color-coded with fluorescent dyes and precoated with analyte-specific antibodies. After magnetic washing, biotinylated detection antibodies and PE-streptavidin were sequentially added. The Attune NxT's red laser identified bead populations while the blue laser quantified PE fluorescence, with concentrations calculated from standard curves.

### 2.8. Statistical analysis

Bioinformatic analysis of gut microbiota was conducted on the Majorbio Cloud Platform through these sequential procedures: (1) α-diversity indices (Observed OTUs, Chao1, Shannon, Good's coverage) and rare faction curves were calculated using Mothur v1.30.2; (2) β-diversity patterns were visualized by principal coordinate analysis (PCoA) based on Bray-Curtis dissimilarity (R vegan v2.4.3 package); (3) treatment effects on community structure were quantified via PERMANOVA (999 permutations); (4) differentially abundant taxa (LDA score > 2, *p* < 0.05) were identified by LEfSe; (5) variance inflation factors (VIF) were computed to address multicollinearity in clinical parameters; (6) distance-based redundancy analysis (db-RDA) with Monte Carlo forward selection (999 permutations) revealed parameter-community relationships, where arrow length in ordination plots indicated explanatory strength; (7) linear regression linked db-RDA-selected parameters with α/β-diversity metrics; (8) microbial co-occurrence networks were reconstructed using Spearman's rank correlation (|ρ| > 0.5, *p* < 0.05). Other experimental data, including survival rate, survival time, body weight, lung weight, cytokines, mRNA levels, viral RNA levels, and lung injury scores, were statistically analyzed using GraphPad Prism 9.3, and all the data are expressed as the mean ± standard deviation (SD). One-way analysis of variance (ANOVA) followed by Tukey's post hoc test for multiple comparisons was performed using GraphPad Prism 9.3 for Windows (GraphPad Software, San Diego California U.S.A., www.graphpad.com), and *p* < 0.05 was considered statistically significant.

## 3. Results

### 3.1. The influence of antibiotic intervention on the mortality of IAV-infected mice

**Figure 2.**
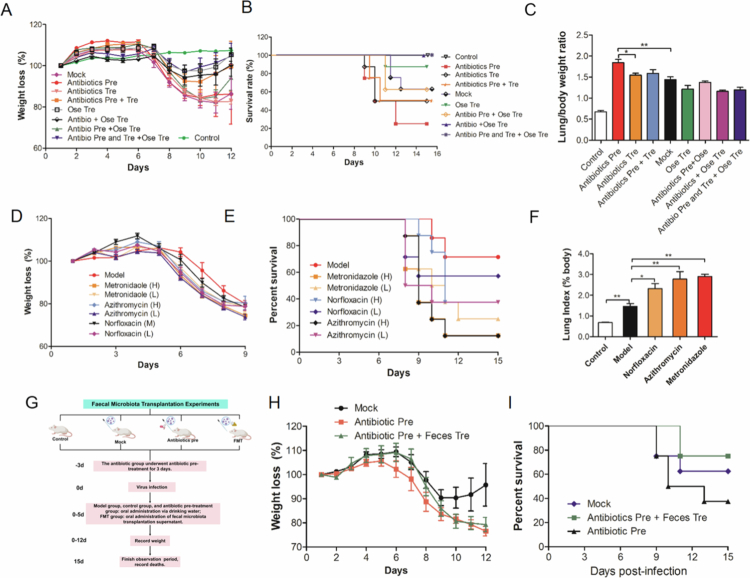
(A) The influence of different antibiotics interventions on the influenza mortality and the efficacy of antiviral drug (oseltamivir), (A) Body weight loss (%), (B) Survival rate (%), (C) Lung/body weight ratio. (B) The influence of different clinical antibiotic pretreatments on influenza mortality; (D) body weight loss (%), (E) survival rate (%), and (F) lung/body weight ratio. (C) Fecal transplantation effectively reverses influenza mortality caused by antibiotic pretreatment. (G) FMT experimental flowchart. (H) Body weight loss (%) and (I) survival rate (%).

The control group exhibited normal weight gain during the observation period and maintained consistent dietary intake. In contrast, the mock group experienced gradual weight loss from day 6 to day 10, followed by gradual recovery (Figure 2A). Deaths in the mock group were first observed on day 11, with a mortality rate of 40% and an average survival time of 13.6 ± 1.9 d (Figure 2B,C). Compared with the mock group, the antibiotic-Pre group showed more pronounced body weight loss, reduced food intake, a prolonged recovery, and shorter survival. This group had a mortality rate of 75% and a survival time of 11.5 ± 2.4 d (Figure 2A–C). The Antibiotics Tre group also exhibited significant reductions in body weight and dietary intake, with a 50% survival rate, which was lower than that of the mock group (40%) but higher than that of the Antibiotics Pre group (75%). Moreover, the survival time of the Antibiotics Tre group (12.4 ± 2.8 d) was significantly shorter than that of the mock group (13.6 ± 1.9 d) but longer than that of the Antibiotics Pre group (11.5 ± 2.4 d) (*p* < 0.05) (Figure 2B,C). To further validate the above findings, we employed the commonly used clinical antibiotics azithromycin, metronidazole, and norfloxacin to investigate the influence of the antibiotic pretreatment on the mortality of IAV-infected mice. Six groups (8 mice/group) were treated with azithromycin, metronidazole, and norfloxacin for 3 d, respectively and then challenged with IAV. Notably, our results revealed that all antibiotic pretreatments exacerbated the mortality of influenza virus-infected mice in a dose-dependent manner by increasing the lung/weight ratio and aggravating weight loss, further confirming the conclusion that antibiotic pretreatment exacerbates death in IAV-infected mice (Figure 2D–F). To determine whether the restoration of the gut microbiota could mitigate the detrimental effects of antibiotic pretreatment during influenza infection, we performed fecal microbiota transplantation (FMT) following viral challenge (Figure 2G). Notably, FMT markedly improved disease outcomes in antibiotic-pretreated mice. In particular, FMT significantly reduced body weight loss (Figure 2H) and effectively reversed the increased mortality caused by antibiotic pretreatment (Figure 2I). Collectively, our results indicated that antibiotic interventions exacerbated mortality in IAV-infected mice (Supplementary Table 2), while restoration of gut microbiota via FMT ameliorated disease severity and improved survival.

### 3.2. The influence of antibiotic intervention on the protective effect of oseltamivir against influenza A virus

Given that antibiotic treatment significantly aggravated IAV-induced mortality, we subsequently assessed whether antibiotic exposure modulates the therapeutic efficacy of antiviral agents. Our results indicated that, compared with the mock group, the Ose Tre group showed significantly lower body weight loss and a reduction in food intake, with body weight returning to its initial levels by day 10 post-infection (Figure 2A). The onset of illness in the Ose Tre group was delayed by 2 d compared with that in the mock group. The mortality of the Ose Tre group (12.5%) was significantly lower than that of the mock group (37.5%), and the survival time of the Ose-Tre group (14.5 ± 1.4 d) was also significantly longer than that of the mock group (13.6 ± 1.9 d) (*p* < 0.05) (Figure 2B,C), confirming the protective effect of oseltamivir against IAV. However, compared with Ose Tre group, antibiotic pretreatment (Antibiotics Pre + Ose Tre) markedly attenuated the protective effect of oseltamivir, manifested as increased mortality (37.5% vs 12.5%), decreased survival time (13.5 ± 2.1 d vs 14.5 ± 1.4 d) (Figure 2B,C), and increased weight loss (Figure 2A). In contrast, compared with the Ose Tre group, the antibiotic treatment (Antibiotics + Ose Tre) exhibited slight higher survival rate (100% vs 87.5%) and slight longer survival time (15 d vs 14.5 ± 1.41 d) than that of the Ose Tre group, indicating that antibiotic treatment has no significant impact on the efficacy of oseltamivir. Furthermore, compared with the Antibiotics Pre + Ose Tre group, the Antibiotics + Ose Tre group and the Antibiotics Pre and Tre + Ose Tre group exhibited higher protective rate (100% vs 62.5%), longer survival time (15 d vs 13.5 ± 2.1 d), and lower weight loss, further confirming the influence of antibiotic pretreatment on the efficacy of antivirals.

### 3.3. The influence of antibiotic intervention on viral lung injury caused by IAV

**Figure 3.**
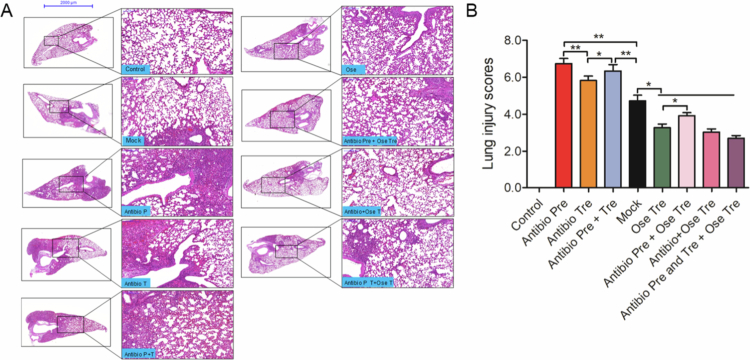
The influence of different antibiotic interventions on acute lung injury caused by IAV. All the treated mice were sacrificed on day 6 post-infection. Lung tissues were collected for histopathological analysis, and the lung injury scores were evaluated with two independently pathologists. Representative images of lung pathological injury (A) and lung injury scores (B).

The lung index is a key indicator for measuring acute lung injury. Therefore, we first investigated the phenotypic differences in the lung indices across all experimental groups. Compared with the control group, all the virus-infected groups presented a significantly increased lung/body weight ratio (Figure 3A) accompanied by severe pulmonary edema, lung consolidation, and hemorrhage. Notably, compared with the mock group, all the antibiotic intervention groups showed more severe pulmonary edema, consolidation, and hemorrhage accompanied by markedly elevated lung/body weight ratios, suggesting that antibiotic treatment exacerbated the lung injury caused by IAV. Notably, the lung/body weight ratio of the antibiotic-pretreated mice were significantly higher than those of both the mock and Antibiotics Tre groups (*p* < 0.05 or *p* < 0.01), suggesting that antibiotic intervention caused more severe lung injury (Figure 3A).

To further assess the effect of antibiotic intervention on IAV-induced acute lung injury, lung histopathological analysis was performed. Compared with the control group, the mock group showed severe lung damage, characterized by a widened pulmonary interstitium, vascular congestion and edema, and many collapsed alveolar cavities with infiltrated lymphocytes and monocytes (Figure 3A,B). However, compared with the mock group, lung tissue injury in all the antibiotic intervention groups was more pronounced, as evidenced by greater widening of the lung interstitium, severe injury to the tracheal wall structure, extensive edema of the alveolar interstitium, and loss of intact alveoli and bronchial lumen structure. Quantitative lung injury scoring further confirmed that damage was more severe in the Antibiotics-Pre group than in the antibiotic-Tre group (Figure 3C). Collectively, these findings demonstrate that antibiotic interventions aggravate lung tissue damage caused by IAV.

### 3.4. The influence of antibiotic intervention on the protective effect of oseltamivir against influenza virus pneumonia

Given that antibiotic interventions exacerbate viral lung injury, we investigated whether antibiotic intervention affects the efficacy of oseltamivir. Compared with the mock group, the oseltamivir-treated group exhibited a good efficacy, as confirmed by reduced lung/body weight ratios (Figure 3A). However, compared with the Ose Tre group, the Antibiotics Pre + Ose Tre group showed higher lung/body weight ratios (Figure 3A,B), whereas the combination of antibiotics and oseltamivir treatment demonstrated slight improvement in the lung/body weight ratios (Figure 3A). In addition, the histopathological analysis results were consistent with the results of the lung/body weight ratio. Compared to the mock group, oseltamivir treatment effectively prevented lung pathological injury caused by IAV. However, compared with the Ose Tre group, the Antibiotics Pre + Ose Tre group showed more severe lung pathological injury, accompanied by severe interstitial edema, alveolar collapse and infiltration of inflammatory cells. In contrast, the Antibiotics Tre + Ose Tre and the Antibiotics Pre and Tre + Ose Tre groups showed slight improvement compared with the Ose Tre group (Figure 3B). Collectively, these findings indicated that antibiotic pretreatment before influenza virus infection markedly attenuated the protective effect of oseltamivir on viral lung injury, whereas antibiotic treatment after infection had no significant influence on viral lung injury caused by IAV.

### 3.5. The influence of antibiotic intervention on excessive inflammatory responses caused by IAV

**Figure 4.**
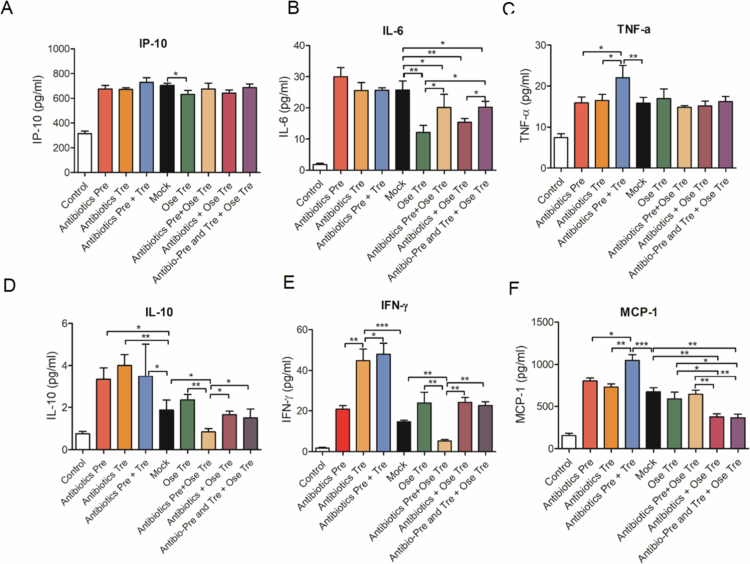
The influence of different antibiotic interventions on excessive inflammatory responses caused by IAV. Mice sera were collected, and the expression levels of inflammatory factors such as IP-10, IL-6, TNF-α, IL-10, IFN-γ and MCP-1 were assessed with Mouse 6-plex flow assay.

Excessive inflammatory responses are central to the clinical severity of influenza infection.^24^^,^^25^ To evaluate whether antibiotic intervention modulates IAV-induced inflammation, the serum levels of key inflammatory cytokines (IP-10, IL-6, TNF-α, IL-10, IFN-γ, and MCP-1) were measured across all antibiotic-treated groups (Figure 4A‒F). Compared with those in the control group, all the treated groups showed markedly increased cytokine levels, suggesting the induction of excessive inflammatory responses by IAV. However, antibiotics exposure shaped these responses in distinct ways. IP-10 levels were not significantly altered among antibiotic-treated groups compared with the mock group, except for a marked reduction in the Ose Tre group. IL-6 levels were higher in the Antibiotics Pre group than in the Antibiotics Tre and the Antibiotics Pre + Tre groups, suggesting that antibiotic pretreatment enhanced inflammatory responses. All oseltamivir-treated groups showed lower IL-6 levels compared with other virus-infected groups; however, this reduction was blunted when antibiotics were administered, likely reflecting the interference of antibiotics with oseltamivir-mediated suppression of viral replication rather than a direct anti-inflammatory effect. The TNF-α levels were elevated in the Antibiotics Pre + Tre group relative to those in the mock, Antibiotics Pre, and Antibiotics Tre groups, indicating an additive effect of prolonged antibiotic exposure in amplifying the hyperinflammatory responses. In contrast, the TNF-α levels were not significantly different between the mock and all the oseltamivir-treated groups. IL-10 and IFN-γ levels were upregulated in all antibiotics-only treated groups compared with mock but their expression levels showed no statistically significant differences among all the Ose-treated groups, except for a marked reduction in the Antibiotics Pre + Ose Tre group. MCP-1 followed a similar pattern, with the Antibiotics Pre + Tre group showing the highest induction, while oseltamivir treatment reduced MCP-1 levels. Notably, this reduction effect caused by oseltamivir treatment was diminished by antibiotic pretreatment but was potentiated by antibiotic therapy.

**Figure 5.**
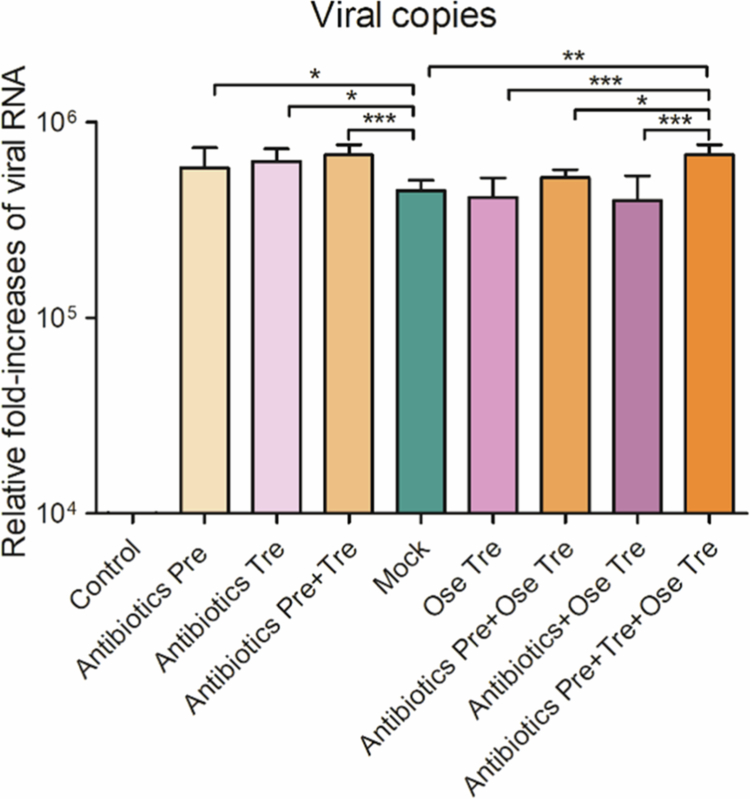
The influence of antibiotic interventions on IAV replication in vivo. The viral RNA levels of all-treated mice on day 6 post-infection were determined by RT-qPCR.

Notably, the integration of these cytokine data with our viral replication measurements (Figure 5) clarifies the underlying mechanism: the lower cytokine levels observed in oseltamivir-treated mice are consistent with reductions in viral copies and are not directly fostered by oseltamivir treatment. Conversely, antibiotic pretreatment and prolonged antibiotic exposure maintained higher cytokine levels despite oseltamivir administration, paralleling the higher viral replication detected in these groups. Thus, impaired antiviral responses, rather than intrinsic immune dysregulation alone, likely drive the amplified inflammatory phenotype in antibiotic-pretreated mice.

Collectively, these findings indicate that antibiotic pretreatment or treatment exacerbates IAV-induced hyperinflammation by upregulating IL-10, IL-6, IFN-γ, TNF-α, and MCP-1. Moreover, antibiotic pretreatment markedly attenuated the beneficial reduction in inflammatory cytokines usually associated with oseltamivir, reflecting its negative impact on viral replication control. Antibiotics treatment alone showed no significant effect on oseltamivir-mediated viral suppression.

### 3.6. Impact of antibiotic intervention on the gut microbiota of influenza virus-infected mice

#### 3.6.1. Effects of different treatments on the richness and diversity of the gut microbiota in mice

**Figure 6.**
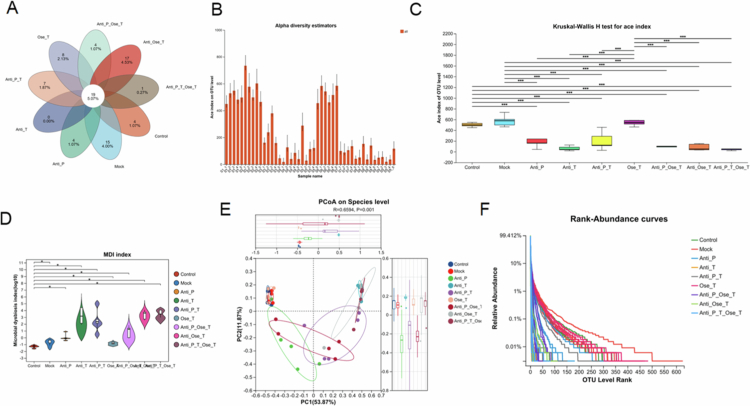
Influence of antibiotic interventions on intestinal microbiota composition and diversity. (A) Venn diagram showing the distribution and overlap of core microbial taxa (OTUs) across experimental groups. Each ellipse represents the number of OTUs detected in a group, and the central intersection indicates the shared core microbiota. (B) ACE index analysis estimating species richness at the OTU level. Bars represent mean ± SD for each group. (C) Comparison of ACE index among treatment groups using the Kruskal–Wallis H test. Box plots display the median, interquartile range (IQR), and outliers. (D) Violin plots illustrating the distribution of the Microbial Dysbiosis Index (MDI) across treatment groups. The width represents data density, the white dot indicates the median, and the vertical bar denotes the IQR. (E) Principal Coordinates Analysis (PCoA) based on Bray–Curtis dissimilarity, showing distinct clustering of gut microbiota at the species level among treatment groups (R = 0.6594, *p* = 0.001, PERMANOVA). PC1 and PC2 explain 53.87% and 11.87% of total variation, respectively. (F) Rank–abundance curves showing relative microbial abundance and evenness for each treatment group. A steeper slope indicates lower evenness, while a longer curve reflects higher richness.

This study integrated Venn diagram analysis, alpha diversity, beta diversity, the Microbial Dysbiosis Index (MDI), and rank-abundance distributions to systematically evaluate how different interventions affect the richness, diversity, and structural organization of the gut microbiota. Venn diagram analysis (Figure 6A) showed that the Antibiotics Pre group shared only four species with the other groups and exhibited no unique species, whereas the Antibiotics + Ose Tre group contained 17 species, indicating broader shifts in microbial composition. Despite these differences, 19 species were shared across all groups, suggesting a relatively stable core microbiota under diverse perturbations.

A comprehensive assessment of alpha diversity demonstrated a clear inhibitory effect of antibiotic exposure on species richness and diversity. ACE index analysis (Figure 6B) revealed that the non-antibiotic groups (Control, Mock, and Ose Tre) maintained markedly higher richness than all antibiotic-related groups, while there was no significant difference between the Control and Mock groups (*p* > 0.05), indicating that IAV infection alone caused only limited disturbance on community composition. Consistent with this, Kruskal‒Wallis analysis (Figure 6C) confirmed significantly lower ACE indices in all antibiotic-treated groups compared with the control group (*p* < 0.001), with the most pronounced reductions observed in the combined antibiotic regimens.

MDI analysis (Figure 6D) corroborated these findings from a homeostasis perspective: MDI analysis (Figure 6D) further supported these findings. The control and mock groups showed the lowest MDI values, indicating relatively balanced communities, whereas all the antibiotic-treated groups showed significantly elevated MDI, which was consistent with aggravated dysbiosis. In contrast, oseltamivir-treated mice showed lower MDI values relative to antibiotics-treated groups, suggesting partial preservation of community balance.

Beta diversity analysis by PCoA (Figure 6E) revealed clear separation in community structure among the treatment groups. The Antibiotics Pre group showed the most distinct clustering pattern, while the Antibiotics Pre + Tre and Antibiotics Pre + Ose Tre groups also demonstrated substantial structural shifts, highlighting the strong impact of early antibiotic exposure on the gut microbiota structure.

Rank‒abundance curves (Figure 6F) further illustrated these patterns. Non-antibiotic groups (Control, Mock, and Ose Tre) showed broader distributions and more even community profiles, whereas all antibiotic-related groups (especially Antibiotics Pre and the combined regimens) showed steeper declines, indicating dominance by a few high-abundance OTUs and substantial loss of low-abundance species. These profiles suggest that antibiotics reduce species richness and promote community imbalance.

Taken together, antibiotic interventions, particularly early or combined exposure, significantly reduced species richness, altered community structure, and increased dysbiosis. In contrast, non-antibiotic interventions were associated with a relatively more balanced community profile. These disruptions provide ecological evidence that antibiotics weaken gut microbial homeostasis, a shift that may contribute to impaired antiviral immunity, aggravate IAV-induced lung injury and undermine antiviral therapy efficacy.

#### 3.6.2. Effects of different treatments on gut microbiota population variability

**Figure 7.**
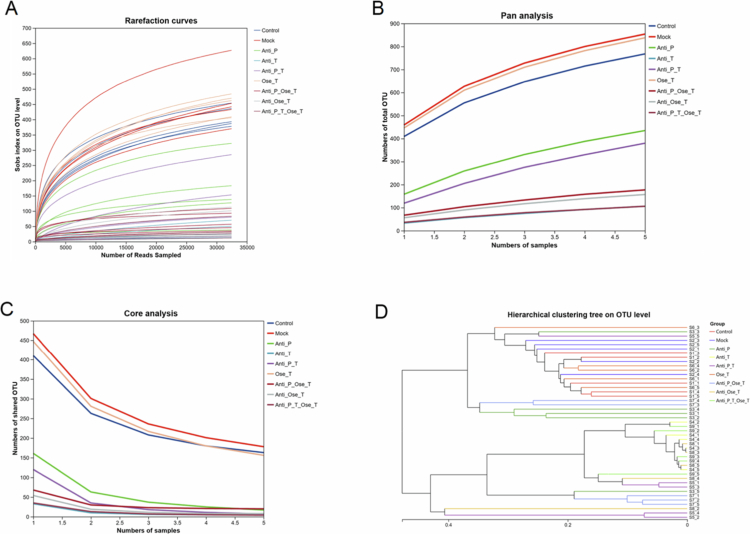
(A) Relationship between sequencing depth and observed OTU counts (Sobs index) across treatment groups. The x-axis denotes the number of subsampled reads per sample, and the y-axis the number of observed OTUs. (B) Rarefaction curves demonstrating total OTU accumulation with increasing sample size. The x-axis indicates sample number, and the y-axis represents the cumulative OTU count. (C) Shared OTU accumulation curves among treatment groups with increasing sample size. The x-axis denotes the number of samples, and the y-axis represents the cumulative number of shared OTUs. (D) Hierarchical clustering dendrogram based on OTU-level similarity between samples. Hierarchical clustering based on OTU-level community composition was performed to examine similarities among all samples. Clustering was calculated using Bray–Curtis dissimilarity metrics. Branch lengths in the dendrogram represent the degree of compositional difference between samples, with shorter branches indicating greater similarity.

To evaluate the influence of different treatments on gut microbiota population dynamics, rarefaction curves and hierarchical clustering analyses were performed (Figure 7). The rarefaction curve revealed distinct patterns of species richness among the treatment groups. Species richness plateaued in the mock and control groups (Sobs index peak: 650 and 580, respectively), indicating stable communities (Figure 7A). In contrast, the Antibiotics Pre and Tre + Ose Tre groups displayed a continued increase in richness beyond this depth (ΔSobs > 150, *p* < 0.01), suggesting the presence of numerous low-abundance, rare taxa. The overall Sobs index followed the gradient Mock > Control > Ose Tre > Antibiotics Tre (*p* < 0.001) while the Antibiotics Pre and Tre + Ose Tre groups exhibited an abnormally high peak (peak: 720), reflecting altered community structure induced by combined treatment (Figure 7A‒C).

Hierarchical clustering using Unweighted Pair Group Method with Arithmetic Mean (UPGMA) based on Euclidean distance (threshold = 0.4) further quantified the structural heterogeneity among groups (Figure 7D). The control and mock groups clustered closely (intergroup distance = 0.12), which was consistent with baseline stability. The Antibiotics Pre and Tre + Ose Tre groups showed the greatest divergence from the mock (distance = 0.35), indicating substantial microbial restructuring and reduced community stability. Although both the Antibiotics Tre and Ose Tre groups were significantly separated from the mock group (distance = 0.25 − 0.3), their clustering patterns suggest distinct modulation pathways by antibiotics versus antiviral agents.

Notably, the diversity gradient revealed by rarefaction curves (Mock > Ose Tre > Antibiotics Tre) aligned with β-diversity findings clustering analysis (Antibiotics Pre and Tre + Ose Tre > Antibiotics Tre ≈ Ose Tre). These complementary results support two key conclusions: (1) antibiotic treatment alone primarily suppresses α-diversity, whereas (2) antibiotics pretreament and treatment combined with oseltamivir treatment exerts a synergistic effect on both α-diversity and β-diversity, driving microbiota communities toward a high-variability, low-stability state that may reflect a new ecological equilibrium.

#### 3.6.3. Effects of different treatments on specific bacterial populations in the mouse gut microbiota

**Figure 8.**
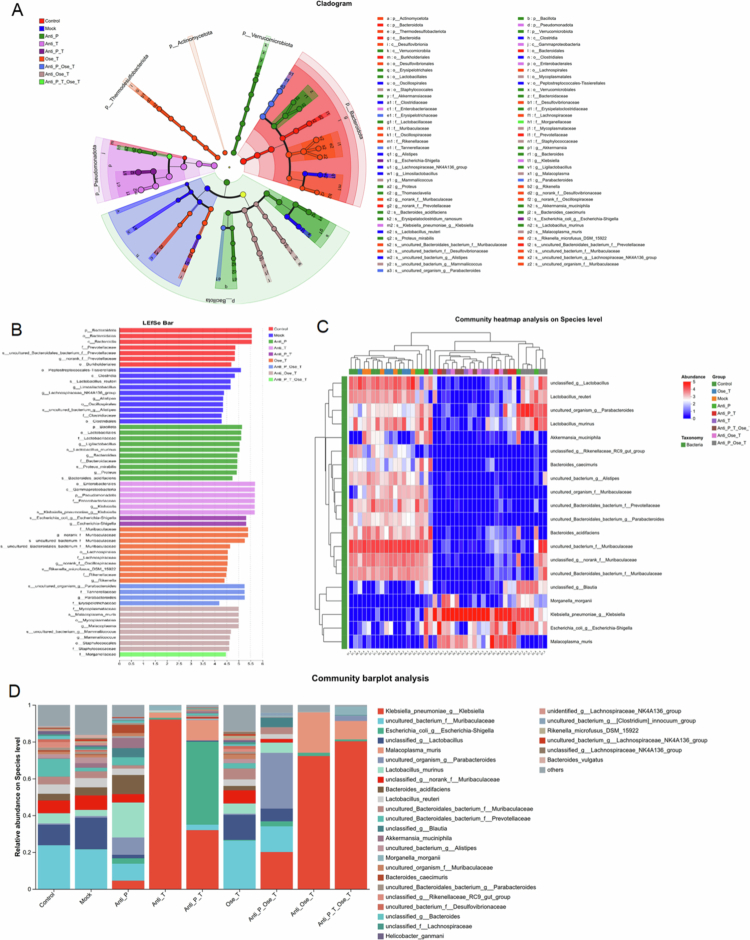
(A) Cladogram generated by linear discriminant analysis effect size (LEfSe), highlighting taxa significantly enriched at different phylogenetic levels (phylum to genus). Colors correspond to specific groups, and each node represents a taxon at a particular phylogenetic level. The size of the node reflects the relative abundance of the taxon, while branch connections indicate phylogenetic relationships. Group-specific coloration identifies taxa predominantly associated with each treatment. (B) Linear discriminant analysis (LDA) scores of differentially abundant taxa identified by LEfSe. Higher scores indicate stronger group-associated enrichment. (C) Heatmap of species-level abundance distribution across treatment groups. Color intensity (blue to red) denotes low to high relative abundance. (D) Stacked bar plot of species-level community composition showing relative abundance of dominant gut microbial taxa across treatment groups.

To assess the impact of different treatment strategies on specific microbial taxa, we performed LEfSe analysis and species-level quantification (Figure 8). Antibiotic interventions significantly altered the gut microbiota composition by depleting sensitive taxa while promoting the proliferation of resistant and potentially pathogenic species. LEfSe analysis revealed that the control group was enriched in high-LDA-score bacterial taxa (LDA > 4.0) across the phylum, class, and order levels, reflecting a stable and diverse microbial community (Figure 8B). In contrast, virus infection alone induced modest reductions in α-diversity and a shift in community structure, characterized by depletion of commensal *Lactobacillus* and *Parabacteroides* lineages and relative enrichment of *Enterobacteriaceae*-affiliated taxa (Figure 8B–D).

The Antibiotics Tre group caused a pronounced loss of α-diversity and selective enrichment of dysbiosis-associated *Pseudomonadota,* with *Klebsiella* being a major discriminator (LDA = 3.5) and a 4.2-fold relative increase compared with control (*p* < 0.001). Beneficial commensals, including *Lactobacillus reuteri*, *Lactobacillus murinus*, and uncultured *Parabacteroides*, decreased by 60%–85% (*p* < 0.05) (Figure 7B–D). Antibiotics Pre amplified these effects, producing lower α-diversity and greater expansion of opportunistic pathogens than Antibiotics Tre alone. The Antibiotics Pre and Tre group produced the most severe dysbiosis, with *Klebsiella pneumoniae* and *Escherichia coli* reaching 6.8-fold and 4.5-fold higher abundances than the control (*p* = 1.89 × 10^−9^ and *p* = 0.007, respectively) (Figure 8C,D).

Oseltamivir alone exerted minimal perturbation on the gut microbiota and partially restored *Lactobacillus*/*Parabacteroides* populations without overt expansion of opportunistic pathogens (Figure 8C,D). However, combined antibiotic and oseltamivir treatment (Antibiotics Tre + Ose) resulted in sustained reductions in α-diversity increased *Klebsiella* and *Escherichia*, and suppressed beneficial commensals (Figure 8B–D). Notably, the Antibiotics Pre and Tre + Ose Tre sequences produced the most simplified community and the highest burdens of *K. pneumoniae* and *E. coli*, (Figure 8C,D) accompanied by marked suppression of *L. reuteri*, *L. murinus*, and *Parabacteroides* (60%–85% reduction, *p* < 0.05), indicating broad disruption of symbiotic populations.

**Figure 9.**
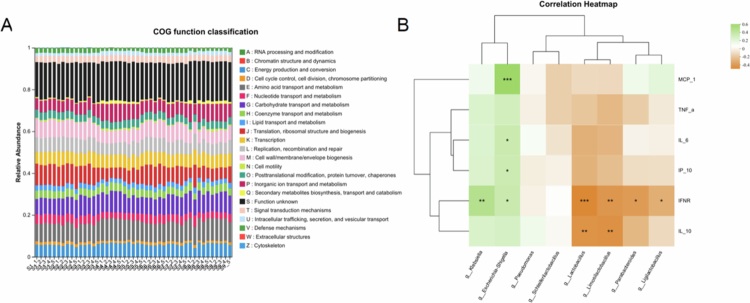
(A) Functional classification of gut microbiota based on COG categories across treatment groups. (B) Heatmap showing correlations between dominant gut microbial genera and inflammatory cytokines. Commensal genera (e.g., g_Lactobacillus, g_Limosilactobacillus, g_Parabacteroides, g_Ligilactobacillus) are negatively correlated with MCP-1, TNF-α, IL-6, and IFN-γ, whereas opportunistic taxa (e.g., g_Klebsiella, g_Escherichia-Shigella) show positive correlations with these cytokines. (*r* ≈ 0.6, *p < 0.05, **p < 0.01, and ***p < 0.001).

Statistical analysis of selected species confirmed the LEfSe and heatmap findings (Figure 8D). One-way ANOVA with Tukey's post hoc test revealed significant group-dependent differences (F(7,40) = 6.72, *p* < 0.001). *Lactobacillus* abundance was markedly reduced in the antibiotic pretreatment and combined treatment groups relative to mock-infected group (*p* < 0.01). *Klebsiella* and *Escherichia–Shigella* were significantly enriched after antibiotic exposure (*p* < 0.001 and *p* = 0.002, respectively), whereas *Parabacteroides* decreased in antibiotic-related groups (*p* = 0.018), consistent with its negative correlation with inflammatory mediators (Figure 9). The Bacteroides abundance did not differ between the oseltamivir-only and control infection groups (*p* = 0.41).

Collectively, these results suggest that antibiotic intervention drives microbial dysbiosis through two primary mechanisms: (1) elimination of sensitive and beneficial taxa, reflected by a 28%–45% reduction in α-diversity, and (2) expansion of resistant and pathogenic bacteria. Combined treatments, particularly Antibiotics Pre and Tre + Ose Tre, exacerbate microbiota simplification via synergistic disruption of community diversity and stability, resulting in a maladaptive microbial equilibrium.

#### 3.6.4. Functional prediction of gut microbiota and its correlation with host inflammatory markers

Functional prediction using PICRUSt-based COG classification showed that core microbial functions related to energy production and conversion, amino acid transport and metabolism, carbohydrate transport and metabolism, translation and ribosomal processes, DNA structure, and replication and repair were conserved across groups, reflecting the baseline metabolic capacity of a healthy gut microbiota (Figure 9A). Mild functional shifts were observed in the virus-infected mock group, including small reductions in energy and carbohydrate metabolism, consistent with modest infection-associated dysbiosis. In contrast, antibiotic exposure produced graded, treatment-dependent suppression of metabolic functions. Both Antibiotics Pre and Antibiotics Tre groups showed reduced amino acid, carbohydrate, and coenzyme metabolism, while the Antibiotics Pre + Tre group exhibited the most pronounced suppression of metabolic functions and enrichment of stress-related pathways, including signal transduction and defense mechanisms, indicating cumulative disruption of microbial metabolic capacity.

Oseltamivir treatment alone (Ose Tre) produced a functional distribution comparable to that of the mock group, implying minimal direct interference with microbial metabolism. However, when oseltamivir was administered after antibiotic exposure (Antibiotics Pre + Ose Tre, Antibiotics Tre + Ose Tre, Antibiotics Pre and Tre + Ose Tre), partial recovery of metabolic functions, particularly in amino acid and carbohydrate metabolism, was observed. The Antibiotics Pre and Tre + Ose Tre groups showed the broadest functional restoration, with enrichment in post-translational modification and inorganic ion transport, suggesting compensatory microbial adaptation under combined antibiotic and antiviral treatment.

To examine how these functional changes are related to host immunity, correlations between key bacterial genera and inflammatory markers were examined (Figure 9B). Antibiotic-induced dysbiosis displayed a bidirectional pattern, marked by the depletion of beneficial taxa and the proliferation of opportunistic bacteria, closely associated with distinct cytokine profiles. *Lactobacillus*-related genera including g_*Lactobacillus*, g_*Limosilactobacillus*, g_*Ligilactobacillus*, g_*Schleiferilactobacillus* were depleted following antibiotic treatment and showed negative correlations with pro-inflammatory cytokines (MCP-1, TNF-α, IL-6, IP-10), consistent with their known SCFA-mediated immunomodulatory roles.^42^^,^^43^ Conversely, opportunistic taxa such as *Escherichia*–*Shigella* and *Pseudomonas* expanded following antibiotics treatment and exhibited strong positive correlations with these cytokines, consistent with their ability to trigger TLR-mediated NF-κB activation and amplify inflammatory responses.^44^

Collectively, these findings demonstrate that antibiotic administration, particularly repeated pre- and post-infection exposure suppresses core microbial metabolic functions and enhances pro-inflammatory host responses. Oseltamivir treatment partially restored microbial functional diversity and improved the microbial-immune balance, highlighting its protective role in mitigating antibiotic-induced perturbation of the pulmonary-intestinal immune axis.

### 3.7. Dysbiosis of gut microbiota homeostasis caused by antibiotics weakens the host's antiviral immunity against IAV in the lung

**Figure 10.**
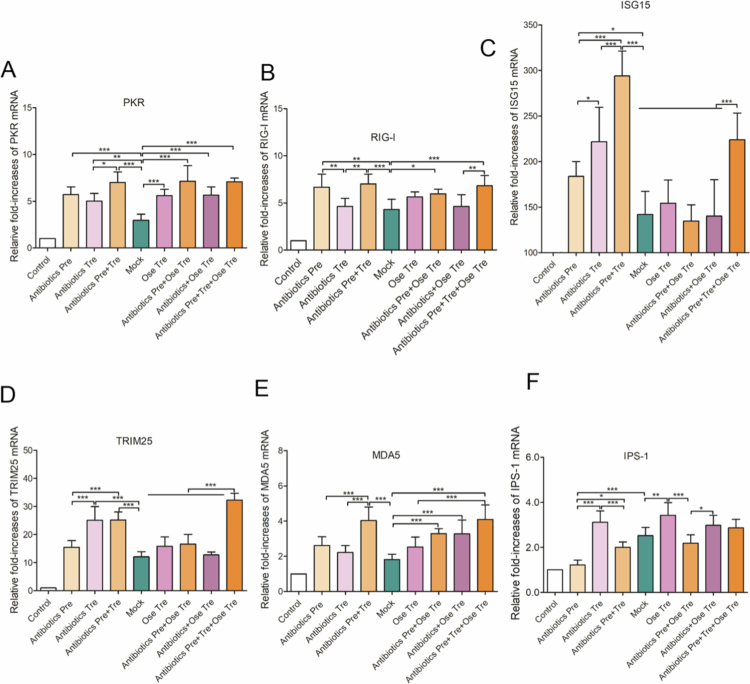
The influence of antibiotic interventions on the host innate antiviral immunity against IAV. RT-qPCR was performed to assess the mRNA levels of host innate antiviral immune factors including PKR (A), RIG-I (B), ISG15 (C), TRIM25 (D), MDA 5 (E), and IPS-1(F).

The RIG-I/IPS-1-mediated antiviral signaling pathway plays a crucial role in both pulmonary and intestinal innate immunity. We first investigated the impact of different antibiotic interventions on the mRNA transcription levels of antiviral immunity factors such as PKR, RIG-I, ISG15, TRIM25, MDA5, and IPS-1. Compared with the control group, all the virus-infected groups presented significant increases in PKR, RIG-I, ISG15, TRIM25, and MDA5 mRNA levels (*p* < 0.05, 0.01, or 0.001) (Figure 9A-F), suggesting that IAV infection activates the pulmonary RIG-I/IPS-1 antiviral signaling pathway. Compared with those in the mock group, the PKR mRNA levels in the Antibiotics Pre, the Antibiotics Tre, and the Antibiotics Pre + Tre groups significantly increased (*p* < 0.05). The PKR mRNA levels in all the oseltamivir-treated groups were also significantly higher than those in the mock group (*p* < 0.001), suggesting that the increase in PKR mRNA levels may be associated with Ose or antibiotic treatment (Figure 10A). In addition, RIG-I mRNA levels in the Antibiotics Pre and Antibiotics Pre + Tre groups were significantly higher than those in both the mock group and the Antibiotics Tre group. Moreover, the RIG-I mRNA levels in the Antibiotics Pre and Tre + Ose Tre group were markedly elevated compared to those in the Antibiotics + Ose Tre group (*p* < 0.01), suggesting that antibiotic pretreatment is the core factor driving the activation of the RIG-I signaling pathway (Figure 10B). In contrast to the RIG-I mRNA expression pattern, the ISG15 and TRIM25 mRNA levels in the Antibiotics Tre and the Antibiotics Pre + Tre groups were significantly greater than those in the Antibiotics Pre group, suggesting that antibiotic treatment after infection may contribute to the upregulation of the ISG15 and TRIM25 mRNAs (Figure 10C-D). ISG15/TRIM25 was also elevated in the repeated antibiotic plus oseltamivir treatment compared to mock and other Ose-treated groups. Additionally, the MDA5 mRNA levels of the Antibiotics Pre group were higher than those in the Antibiotics Tre group (*p* < 0.05) but significantly lower than those in the Antibiotics Pre + Tre group (*p* < 0.001), whose levels markedly increased relative to those in the mock group. These findings suggest that antibiotic pretreatment and treatment may synergistically increase MDA5 expression levels (Figure 10E). The MDA5 mRNA levels in all the oseltamivir and antibiotic combination groups were significantly higher than those in the mock group. Notably, the MDA5 mRNA levels in the Antibiotics Pre and Tre + Ose Tre groups were markedly higher than those in both the mock and Ose Tre groups, further indicating that antibiotics intervention contributes to the upregulation of MDA5 mRNA. In contrast, the expression profile of IPS-1 mRNA differed from that of the other antiviral signaling genes. Specifically, IPS-1 mRNA levels in the Antibiotics Pre group were not only markedly lower than those in the Antibiotics Tre group and the mock group (*p* < 0.001) but also significantly lower than those in the Antibiotics Pre + Tre group (*p* < 0.05), suggesting that antibiotic pretreatment strongly suppresses IPS-1 expression (Figure 10F). Furthermore, while IPS-1 mRNA levels were significantly elevated in the Ose Tre group relative to those in the mock group, they were markedly reduced in the Antibiotics Pre + Ose Tre group compared to the Ose Tre group. These findings suggest that antibiotic pretreatment may impair IPS-1-dependent antiviral signaling pathways, potentially dampening the host's innate immune response. To investigate whether the observed modulation of antiviral signaling pathways correlates with viral replication, we quantified viral loads across all virus-infected groups. Viral loads were significantly increased in the Antibiotics Pre, Antibiotics Tre, and Antibiotics Pre + Tre groups compared to the mock group (*p* < 0.05 or 0.001), suggesting that antibiotics intervention may promote viral replication (Figure 5). However, the Antibiotics Pre + Tre and Antibiotics Pre and Tre + Ose Tre groups exhibited significantly higher viral loads than the mock and Ose Tre groups. These findings provide additional evidence that antibiotic intervention promotes viral replication. Collectively, our results indicate that antibiotic intervention not only disrupts the host's antiviral immune homeostasis, as evidenced by increased mRNA levels of PKR, RIG-I, ISG15, and TRIM25 alongside reduced IPS-1 expression, but also promotes viral replication. This dual impact may represent a primary mechanism by which antibiotic intervention exacerbates influenza-associated mortality and lung injury. Furthermore, the antibiotic-induced imbalance of RIG-1/IPS-1 signaling pathways may significantly impair the protective efficacy of oseltamivir.

### 3.8. Dysbiosis of gut microbiota homeostasis caused by antibiotics weakens the host's antiviral response to influenza a virus in the gut

**Figure 11.**
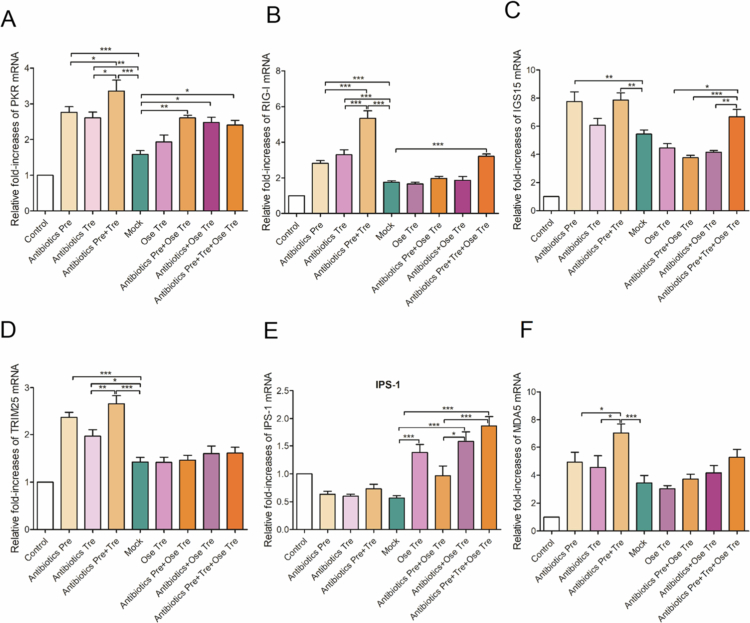
The influence of antibiotic intervention on the host innate antiviral immunity against influenza A virus in intestinal tissue. RT-qPCR was performed to assess the mRNA levels of host innate antiviral immunity factors, including PKR (A), RIG-I (B), ISG15 (C), TRIM25 (D), IPS-1 (E), and MDA5 (F).

Given that antibiotic-induced dysbiosis attenuates antiviral immunity in the lungs, we further investigated whether antibiotics affect the host's antiviral immunity by regulating the intestinal microbiota. Compared with those in the control group, the intestinal PKR mRNA levels were significantly increased in all the virus-infected groups (*p* < 0.01 or 0.001) (Figure 11A), indicating the activation of the intestinal PKR signaling pathway upon IAV infection. Importantly, all three antibiotic-treated groups showed markedly increased intestinal PKR mRNA levels compared to the mock group (*p* < 0.05, 0.01, or 0.001), with the Antibiotics Pre + Tre group showing the highest expression. This pattern suggests a cumulative activating effect of antibiotic intervention on the intestinal PKR signaling pathway. In contrast, the PKR mRNA levels in the Ose Tre group were only slightly higher than those in the mock group (*p* > 0.05). Notably, the PKR mRNA levels in the Antibiotics Pre + Ose Tre, Antibiotics + Ose–Tre, and the Antibiotics Pre and Tre + Ose Tre groups were significantly higher than those in the mock group (*p* < 0.05, or 0.01), further supporting the association between antibiotic intervention and the upregulation of PKR mRNA. Consistent with the PKR expression patterns, the RIG-I mRNA levels were significantly elevated in all virus-infected groups (*p* < 0.05 or 0.001) (Figure 11B), suggesting that influenza virus infection also activates the RIG-I signaling pathway in the gut. Notably, RIG-I mRNA levels in the Antibiotics-Pre, Antibiotics-Tre, and Antibiotics Pre + Tre groups were markedly higher than those in the mock group (*p* < 0.01 or 0.001), suggesting that antibiotic exposure may synergistically potentiate IAV-induced activation of the intestinal RIG-I signaling pathway. However, the intestinal RIG-I mRNA levels in the Ose Tre, Antibiotics Pre + Ose Tre, and Antibiotics Tre + Ose Tre groups were comparable to those in the mock group but significantly lower than those observed in the Antibiotics Pre and Tre + Ose Tre groups. These findings suggest that oseltamivir treatment alone does not affect the RIG-I signaling pathway, whereas combined antibiotic pretreatment contributes substantially to its upregulation. In addition, ISG15 mRNA levels were significantly elevated in all the virus-infected groups compared to the control group, indicating that influenza virus infection induces ISG15 expression in the gut (Figure 11C). Notably, the ISG15 mRNA levels in the Antibiotics Pre group and the Antibiotics Pre + Tre group were significantly higher (*p* < 0.01). In contrast, no significant difference was observed between the Antibiotics-Tre group and the mock group. These results suggest that the activation of ISG15 is driven primarily by antibiotic pretreatment. Furthermore, the ISG15 mRNA levels in the Ose Tre, the Antibiotics Pre + Ose Tre, and Antibiotics + Ose Tre groups were slightly lower than those in the mock group, while the Antibiotics Pre and Tre + Ose Tre group exhibited markedly elevated ISG15 expression compared to the other Ose-treated groups. These findings further confirm the critical role of antibiotic pretreatment in promoting the upregulation of ISG15. Regarding TRIM25 mRNA expression, all the antibiotic pretreatment groups exhibited an upward trend, with statistically significant increases (*p* < 0.001 or 0.01), suggesting that TRIM25 may play a role in the amplification of RIG-I/IPS-1-mediated antiviral signaling (Figure 11D). In contrast, IPS-1 mRNA levels were significantly lower in the Antibiotics Pre and Tre + Ose Tre, Ose Tre, and Antibiotics Tre + Ose Tre groups compared to the control (*p* < 0.05) (Figure 11E). The other groups also showed varying degrees of IPS-1 reduction. Importantly, IPS-1 mRNA levels in the Antibiotics Pre + Tre group were slightly elevated relative to those in the Antibiotics Tre group. In contrast, a marked downregulation was observed in the Antibiotics Pre + Ose Tre group compared to the Ose Tre group. These findings suggest that antibiotic pretreatment may impair IPS-1-dependent antiviral signaling, potentially inhibiting innate immune responses to influenza virus infection. Compared with the corresponding controls, all antibiotic pretreatment groups demonstrated significant upregulation of MDA5 mRNA transcription (*p* < 0.001 or 0.01), suggesting that antibiotic intervention may enhance MDA5-dependent antiviral signaling (Figure 11F).

Integrated analysis of the transcriptional profiles of key intestinal antiviral signaling mediators alongside viral replication levels revealed a coordinated upregulation of PKR, RIG-I, ISG15, TRIM25, and MDA5 mRNA levels, accompanied by a notable downregulation of IPS-1 mRNA. These transcriptional patterns, which are consistent with those observed in the lungs, suggest that antibiotic pretreatment activates the PKR pathway and promotes IFN-1 expression, potentially exacerbating inflammatory responses. Concurrently, disruption of the RIG-I/IPS-1 antiviral signaling pathway, characterized by reduced IPS-1 expression, may weaken viral suppression, ultimately compromising the efficacy of antiviral therapies such as oseltamivir.

## 4. Discussion

**Figure 12.**
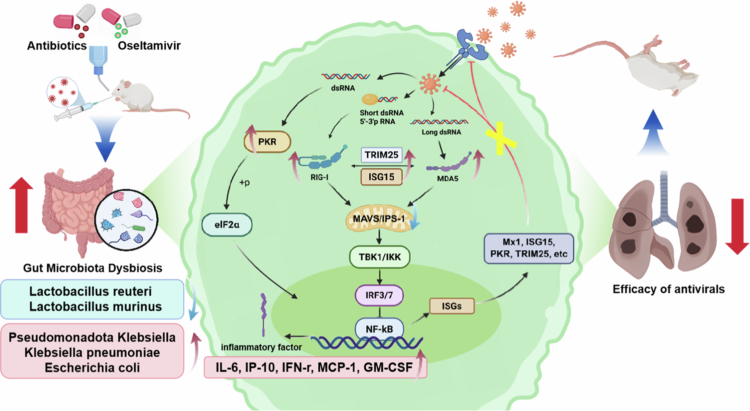
Proposed mechanism by which antibiotic-induced gut microbiota dysbiosis exacerbates mortality during IAV infection.

In this study, we investigated the impact of antibiotic-induced gut dysbiosis on host antiviral immunity and the efficacy of oseltamivir against influenza virus using an H1N1-infected mouse model. Our findings demonstrated that antibiotic intervention significantly disrupted the gut microbiota composition and function, weakened host antiviral immune responses, and reduced the effectiveness of antiviral therapy (Figure 12). Notably, our results also suggest that the combination of antibiotics and antivirals may partially restore immune imbalance in antibiotic-pretreated hosts, providing a more comprehensive perspective on combination strategies.

Consistent with prior studies,^38^^,^^45^ fecal microbiota profiling revealed a marked decrease in microbial richness and diversity following antibiotic intervention. LEfSe analysis further indicated that antibiotics selectively depleted beneficial genera, such as *Lactobacillus*, while enriching drug-resistant and opportunistic pathogens, including *K. pneumoniae* and *E. coli*. These changes reflect the classical features of gut dysbiosis. Importantly, dysbiosis was associated with increased mortality, shortened survival time, and aggravated lung injury in antibiotic-treated mice, even in the presence of antiviral therapy. These results underscore the role of a balanced gut microbiota in supporting mucosal immunity and enhancing antiviral defense.

Mechanically, the viral RNA produced during viral invasion of host cells or viral replication can promote the expression of the upstream viral RNA sensors RIG-I/PKR, which will activate RIG-I/MDA5 signaling and promote the expression of IPS-1 (a key adaptor for downstream type I interferon signal transduction), thereby promoting interferon production through downstream pathways and facilitating viral clearance. Theoretically, only blocking this antiviral signaling pathway in target cells for virus replication may compromise the host's antiviral defense. Because the intestines are not target organs for influenza virus invasion, it rarely occurs to anyone that there might be a connection between the gut and the antiviral immunity. In this study, our results revealed for the first time that antibiotic-induced dysbiosis, the expression of the upstream viral RNA sensors RIG-I and PKR was upregulated in both lung and intestinal tissues, while the expression of IPS-1 (also known as MAVS) was significantly downregulated (Figure 12). Previous studies have indicated that IPS-1 reduction can arise via multiple, non-mutually exclusive mechanisms: host-mediated ubiquitination and proteasomal turnover driven by PCBP2 recruitment of the HECT-type E3 ligase AIP4^46^; direct proteolytic cleavage by viral proteases, which displace or fragment mitochondrial IPS-1^47^; functional inhibition by viral accessory proteins that block IPS-1–RIG-I interactions^48^; infection-induced microRNAs that repress IPS-1 transcripts^46^; and apoptosis-associated caspase cleavage of IPS-1.^47^ Each of these processes converges on the loss of functional IPS-1 and thereby disrupts the formation of the mitochondrial signaling platform required for TBK1-IRF3 and IKK-NF-κB activation. The consequence is impaired IFN-β induction and broadly weakened RLR-mediated antiviral signaling,^46^^,^^47^ which is expected to facilitate viral replication and exacerbate tissue pathology. This imbalance in the expression of key components of the antiviral signaling pathway may lead to a diminished or even abolished antiviral response. Consequently, the affected cells and tissues exhibited prominent pathological features, including pulmonary interstitial edema, alveolar collapse, and inflammatory cell infiltration (Figure 3B). our results indicate that antibiotic-induced dysbiosis impairs innate antiviral immunity through disruption of the IPS-1-dependent signaling cascade (Figure 9).

Given that IPS-1 is a critical adaptor linking RIG-I/MDA5 activation to downstream type I interferon signaling, its suppression suggests a key impairment in the antiviral signaling pathway that limits interferon production and viral clearance.^49^^,^^50^ This finding aligns with previous reports that identified IPS-1 as essential for IRF3/7 and NF-κB activation via TBK1 and IKKε, and that its deficiency impairs host defense against RNA viruses.^49^

Our observations are supported by the work of Erttmann et al., who demonstrated that gut commensals maintain systemic antiviral competence through cGAS-STING-IFN-I signaling, and that antibiotics suppress tonic interferon responses.^51^ Similarly, Abt et al. demonstrated that microbiota depletion raises the activation threshold of macrophage antiviral responses, while Bradley et al. showed that an intact gut microbiota sustains tonic type I interferon signaling in lung stromal cells, thereby maintaining an antiviral state that restricts early influenza virus replication and disruption of this signaling compromises early viral control.^52^^,^^53^ In addition, recent studies have increasingly highlighted the importance of the flu vaccine in preventing influenza. For instance, Oh et al. reported that a trivalent inactivated vaccine (TIV) does not directly activate signaling through Toll-like receptor 5 (TLR5)^54^; instead, the intestinal microbiota plays a facilitating role in promoting the TLR5-mediated immune response to TIV. A review of the existing literature reveals that both clinical studies and animal models suggest that the complex composition of the gut microbiota is a key regulator of the immune response following vaccination.^55^ Furthermore, research by Hagan and colleagues has provided evidence that antibiotic-induced disruption of the microbiome may influence the immune response to vaccination in healthy adults.^56^ These findings reinforce the role of the microbiota in priming antiviral immunity. Clinically, antibiotics are not recommended for viral infections owing to their lack of efficacy against viruses.^57^ However, few studies have addressed how prior antibiotic exposure affects subsequent responses to viral infection and antiviral treatment. In recent years, the lung–gut axis, recognized as a crucial bidirectional regulatory network linking the respiratory system with the intestinal microecosystem, has emerged as a focal point in immunoregulatory research. An emerging study reported that the gut microbiota may influence the immune responses of distant organs, including the lungs, through modulation of the mucosal immune system.^58^ In addition, during vaccination, the composition and diversity of the gut microbiota may influence the maturation of dendritic cells and subsequent T cell responses via microbial metabolites (e.g., short-chain fatty acids) or signaling pathways (e.g., IPS-1 and TLR), thereby modulating both systemic and mucosal immune responses.^59^^,^^60^ Our results provide novel evidence that prior antibiotic treatment weakens antiviral immunity and reduces the efficacy of oseltamivir. This was reflected in poorer survival outcomes, enhanced lung pathology, and altered immune gene expression in antibiotic-pretreated mice receiving oseltamivir. These observations are consistent with clinical data from Hagan et al., who reported that antibiotics impaired influenza vaccine responses in individuals with low baseline immunity, associated with increased inflammatory gene signatures and reduced bile acid levels.^61^ Therefore, we supposed that the observed imbalance of immune responses in the antibiotic-treated mice in this study may be partially attributable to the modulatory effects of the gut microbiota on the host's antiviral immunity responses via the lung‒gut axis.

Interestingly, we observed that the combination of antibiotics and antivirals improved survival and immune responses primarily when antibiotics were administered after infection, even in mice that had received antibiotics prior to infection. This contrasts with the conventional view that antibiotics are contraindicated in viral infections and aligns with clinical observations where combination therapy improved recovery in patients with severe influenza.^62-64^ Additionally, large-scale data from a Japanese cohort suggest that antibiotic‒antiviral coprescription may benefit particular subgroups, such as older adults, although broader use requires careful consideration.^65^ Our microbiota analysis revealed that combination therapy partially restored microbiota structure and function, shifting it toward a new equilibrium with altered diversity. The improved therapeutic efficacy may be attributable to the restoration of the IPS-1 (MAVS) signaling pathway, the inhibition of pulmonary inflammation, and viral replication. However, the precise mechanisms remain to be elucidated in the future.

While our study provides novel mechanistic and translational insights, it has limitations. First, the findings in mice require validation in human studies. Second, the role of microbial metabolites in modulating IPS-1 signaling remains unexplored. Third, although we characterized key immune genes, a more comprehensive multiomics analysis could further enhance the mechanistic understanding. Finally, the long-term effects of antibiotic‒antiviral combination therapy on microbiota stability and host immunity have not been assessed. Furthermore, this study was conducted using a mouse model, and the findings cannot be directly extrapolated to humans.

In conclusion, this study demonstrated that antibiotic-induced dysbiosis compromises host antiviral immunity and reduces oseltamivir efficacy, largely through the impairment of IPS-1-mediated signaling. Importantly, combination therapy may offer immunological benefits in antibiotic-pretreated hosts, warranting further investigation. These findings emphasize the critical role of the gut–lung immune axis in viral infection and offer a framework for optimizing therapeutic strategies in patients with recent antibiotic exposure.

## Supplementary Material

Supplementary_Material.docx Supplementary_Material.docx

## Data Availability

16S rRNA gene sequencing data have been deposited in the SRA database and can be assessed via https://www.ncbi.nlm.nih.gov/sra/PRJNA1274617.
