# Supplementary material for: The potential immunological mechanisms of gut microbiota dysbiosis caused by antibiotics exacerbate the lethality of influenza viruses
Source: Gut Microbes. 2026 Jan 2;18(1):2609451. doi: 10.1080/19490976.2025.2609451 (PMC12773635; doi:10.1080/19490976.2025.2609451)
Supplement: Supplementary_Material.docx [file KGMI_A_2609451_SM8825.docx]

| Gene name | Primer sequences |
| --- | --- |
| mMDA5 F | TGTGATTGCTGATGACACCAGA |
| mMDA5 R | CCACTGCTCATAATGTTGGGTTC |
| mIPS-1 R | CTAGCAACCTATGGACCTGTGT |
| mIPS-1 F | AAAGCTGATCCAGTGGACGAG |
| mISG15 F | CCACAGCAACATCTATGAGGTC |
| mISG15 R | CGCAAATGCTTGATCACTGT |
| mPKR F | ATGGTACTGGTTCAGGTGTCAC |
| mPKR R | TGTCATAGACGAGCTGCTGGA |
| mRIG-I F | CCAGAGTGTCAGAATCTCAGTCAG |
| mRIG-I R | GGCAAGCTCCAGTTGGTAATT |
| mTRIM5 F | ACTGAAGGCAGAGGTTGAGC |
| mTRIM5 R | CTATCTTAGGGATGTAGACTGGTTT |

**Supplementary Table 1**

**Supplementary Table 2**

| Groups | Mice (n) | Death (n) | Survival (n) | Mortality (%) | Survival time (days) | Life extension rate (%) |
| --- | --- | --- | --- | --- | --- | --- |
| Control | 8 | 0 | 8 | 0 | 15.0±0.0 | 100 |
| Model | 8 | 3 | 5 | 37.5 | 13.6±1.9 | 0 |
| Antibio Pre | 8 | 6 | 2 | 75** | 11.5±2.5 | -154.5*** |
| Antibio Tre | 8 | 4 | 4 | 50* | 12.4±2.8 | -90.9** |
| Antibio Pre + Tre | 8 | 4 | 4 | 50* | 12.3±3.0 | -100*** |
| Ose Tre | 8 | 1 | 7 | 12.5** | 14.5±1.4 | 63.6** |
| Antibio Pre + Ose Tre | 8 | 3 | 5 | 37.5 | 13.5±2.1 | -9.1* |
| Antibio Tre + Ose Tre | 8 | 0 | 8 | 0*** | 15.0±0.0 | 100*** |
| Antibio Pre and Tre + Ose Tre | 8 | 0 | 8 | 0*** | 15.0±0.0 | 100*** |

Note：^*^*P*<0.05，^**^*P*<0.01，*^***^P*<0.001 indicated these treated groups vs the mock.
